# Efficacy of the Quorum Sensing Inhibitor FS10 Alone and in Combination with Tigecycline in an Animal Model of Staphylococcal Infected Wound

**DOI:** 10.1371/journal.pone.0151956

**Published:** 2016-06-02

**Authors:** Oriana Simonetti, Oscar Cirioni, Ivana Cacciatore, Leonardo Baldassarre, Fiorenza Orlando, Elisa Pierpaoli, Guendalina Lucarini, Elena Orsetti, Mauro Provinciali, Erika Fornasari, Antonio Di Stefano, Andrea Giacometti, Annamaria Offidani

**Affiliations:** 1 Clinic of Dermatology, Department of Clinical and Molecular Sciences; Università Politecnica delle Marche – Ospedali Riuniti, Ancona, Italy; 2 Clinic of Infectious Diseases, Italy, Department of Biomedical Sciences and Public Health; Università Politecnica delle Marche – Ospedali Riuniti, Ancona, Italy; 3 Department of Pharmacy, Università degli Studi G. D’Annunzio, Chieti-Pescara, Italy; 4 Experimental Animal Models for Aging Units, Research Department, I.N.R.C.A. I.R.R.C.S., Ancona, Italy; 5 Clinic of Dermatology, Department of Clinical and Molecular Sciences; Università Politecnica delle Marche, Ancona, Italy; Central South University, CHINA

## Abstract

In staphylococci, quorum sensing regulates both biofilm formation and toxin production, moreover it has been demonstrated to be inhibited by RNAIII inhibiting peptide (RIP). Aim our study was to evaluate the *in vitro* activity and its *in vivo* efficacy of the combined administration of FS10, a novel RIP derivative, and tigecycline in an animal model of methicillin-resistant (MR) and methicillin-sensitive (MS) *Staphylococcus aureus* wound infection. Using a 1.x2 cm template, one full thickness wound was established through the panniculus carnosus on the back subcutaneous tissue of each animal. Infection was determined by inoculation of 5x10^7^ CFU/ml of bacteria, that produced an abscess within 24 h, after this, treatment was initiated. The study included, for each strain, a control group without infection, a control infected group that did not receive any treatment and a control infected group with drug-free foam dressing, and three infected groups treated, respectively, with: FS10-soaked foam dressing (containing 20 μg FS10), daily intraperitoneal tigecycline (7 mg/Kg), FS10-soaked foam dressing (containing 20 μg FS10) and daily intraperitoneal injections of tigecycline (7 mg/Kg). The main outcome measures were quantitative culture and histological examination of tissue repair. The highest inhibition of infection was achieved in the group that received FS10-soaked and parenteral tigecycline reducing the bacterial load from 10^7^ CFU/ml to about 10^3^ CFU/g for MSSA and to about 10^4^ CFU/g for MRSA. The group treated with FS10-soaked foam dressing associated with parenteral tigecycline showed, histologically, better overall healing with epithelialization and collagen scores significantly higher than those of the other groups in both strains. In conclusion, the combined use of topical FS10 with i.p. tigecycline induced positive interaction *in vivo*, resulting in an enhanced therapeutic benefit versus staphylococcal infections in murine wound models.

## Introduction

*Methicillin-resistant Staphylococcus aureus* (MRSA) has emerged as an important cause of hospital- and community-acquired infections [1]. Recent data on the epidemiology of *S*. *aureus* indicate that epidemical methicillin-resistant *S*. *aureus* (MRSA) strains have increased in virulence representing a significant threat to public health because of multidrug resistance and strong biofilm- forming properties. Biofilms are adherent communities of bacteria embedded in a self-produced extracellular polymeric matrix [2–3]. Adaptation to surface attached growth within a biofilm is accompanied by significant changes in gene and protein expression, as well as by metabolic activity. Coordination between the different bacteria occurs through a mechanism of cell-to-cell communication called quorum sensing (QS) [4].

In common to both community- and hospital-associated *S*. *aureus* infections, antibiotics resistance is an increasing problem, therefore there is a compelling need to develop novel and effective classes of antibiotics to counteract the drug-resistant wound isolates [5]. Antimicrobial peptides (AMPs) are integral components of the innate host defense mechanism in many organisms, such as plants, insects, amphibians, and mammals. AMPs are emerging as a promising new generation of antibiotics because of their rapid and broad-spectrum antimicrobial properties, their ability to kill multidrug-resistant bacteria, and their low propensity for developing resistance [6–7]. Moreover they act efficiently and rapidly against a wide range of pathogens from bacteria, to fungi, yeasts, viruses and protozoa [8–13].

In staphylococci, quorum sensing regulates both biofilm formation and toxin production, and it has been demonstrated to be inhibited by the RNAIII inhibiting peptide (RIP) [14–18]. Recently, we synthesized novel RIP derivatives (FS1-11) to identify the smallest active sequence endowed with antistaphylococcal activity [19]. Our outcomes showed that FS3, FS8, and FS10 were found to be significantly more active than RIP [20–21].

**Fig 1:**
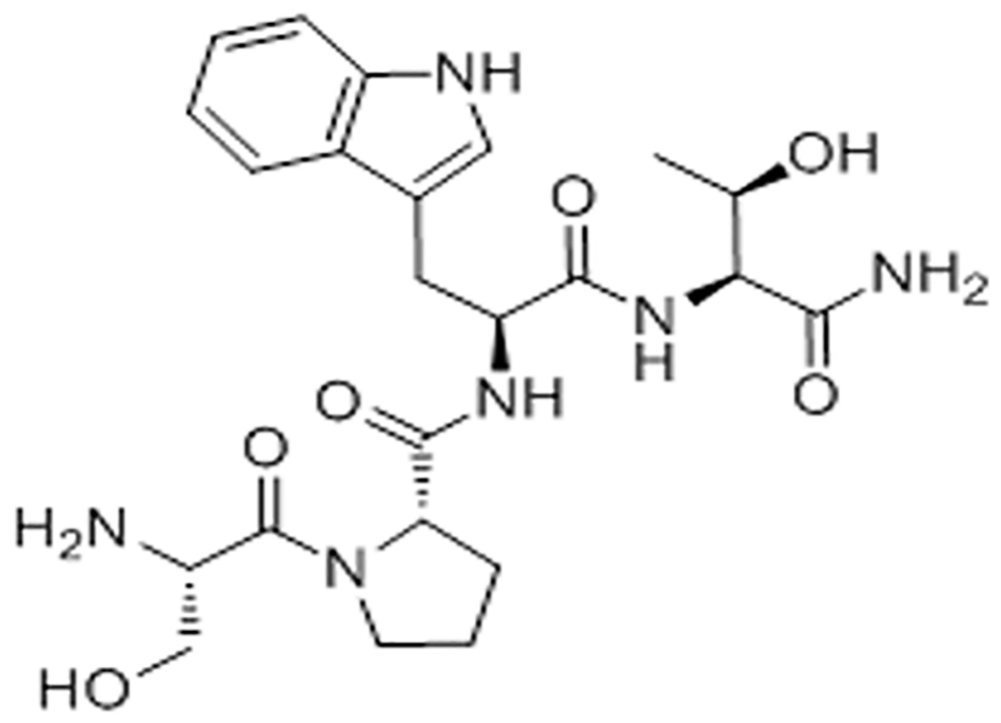
Chemical structure of FS10.

Notably, FS10 - corresponding to the linear sequence H-Ser-Pro-Trp-Thr-NH_2_ (Fig 1) - is a tetrapeptide containing the residues of proline in P2 and threonine in P4. We observed that these residues, common to the sequence of RIP and RNAIII activating peptide (RAP), are essential for the inhibition of staphylococcal bacterial infection [19]. Probably, the favorable distance between hydroxyl groups of serine and threonine, acting as hydrogen donors or acceptors, and the aromatic moiety of triptophan could interfere with the function of *S*. *aureus* quorum sensing, reducing bacterial pathogenicity. In fact, although the mechanism of action has yet to be elucidated, FS10 could be a molecule that mimics autoinducer structure interfering with the stability and function of the regulator protein or the autoinducer synthase [22].

Tigecycline is the first drug in the glycylcycline class, a new class of antibiotics derived from tetracycline [23] that exhibits potent activity against a broad spectrum of bacteria, including staphylococci. Recently a synergistic effect was reported when tigecycline was combined with other clinically-used antibiotics or antimicrobial peptides [8, 24–25].

The aim of the present study was to evaluate the *in vivo* efficacy of the combined administration of FS10 and tigecycline in an animal model of staphylococcal wound infection.

## Materials and Methods

### Organisms

The quality control strains methicillin-resistant (MR) *S*. *aureus* ATCC 43300 and methicillin-sensitive (MS) *S*. *aureus* ATCC 29213 were used (Becton Dickinson Italia, Milan, Italy).

### Animals

Adult male BALB/c mice weighting 35 to 50 g were used for all the experiments. All animals were housed in individual cages under constant temperature (22°C) and humidity, with 12-hour light/dark cycle, and had access to chow and water as much as desired throughout the study. Procedures and facilities followed the requirements of Commission Directive 86/609/EEC concerning the protection of animals used for experimental and other scientific purposes. Italian legislation is defined in D.L. no. 116 of January 27, 1992. Experimental protocols were also approved by the Institutional Animal Care Committee of the Ministry of Health (Italy) and by the Animal Research Ethics Committee of IRCCS-INRCA.

### Drugs

FS10 was synthesized as previously reported by us [19]; briefly, we used solid phase synthesis via Fmoc chemistry on a rink amide resin. The purity of FS10 was analyzed by reverse phase HPLC (Waters Co., Milford, MA, USA) using an X-Bridge BEH130 C18, 5 mm, 4.6 x 250 mm column and a Waters 2996 PDA detector (280 nm and 220 nm) while its molecular weight was confirmed by mass spectrometry using a LC-MS/MS system consisting of an LCQ (Thermo Finnigan, San Jose, CA, USA) ion trap mass spectrometer equipped with an electrospray ionization (ESI) source.

Tigecycline (Pfizer Italia, Aprilia (LT), and FS10 were diluted in accordance with manufacturers’ recommendations yielding 10 mg/mL stock solutions. Solutions were made fresh on the day of assay or stored at -80°C in the dark for short periods.

### Absorbance and elution profiles of FS10 by Allevyn

We used Allevyn (Smith & Nephew Healthcare, St Hull East Riding of Yorkshire,UK) [26], a foam wound dressing, which can be soaked in an antistaphylococcal agent. Pieces of Allevyn (1x2 cm^2^) were placed in 20 ml FS10 (10 μg/mL). Allevyn slices were soaked at room temperature in FS10 solution, in triplicate, for 30 min (1 mL of saline per graft, containing 10 mg L^-1^ of FS10), to determine the peptide concentration impregnated on Allevyn. Following the incubation period (20 min), the Allevyn was removed, and the peptide concentration in solution was determined using reverse-phase HPLC. The unbound peptide concentration was calculated by area integration of the UV-absorbing peak (220 and 280 nm) and compared with standard curves of the reference peptide [20]. Allevyn pieces were placed in 5ml water to determine elution profile where 1 ml solution was removed after 2, 5 and 24 hrs. One ml of unabsorbed and eluted fraction was applied to a high pressure liquid chromatography (HPLC) reverse phase column (Thermo Elecron Corp, Hypersil Gold 150 x 4.6 mm) and eluted by a 0–70% acetonitrile in 0.1% TFA gradient at a flow rate of 1 ml/min. The peptide, that eluted at about 50% acetonitrile, was detected spectroscopically at 216nm or 280nm. It is noteworthy that, within minutes, 2 ml FS10 solution was absorbed by the Allevyn 1x2cm^2^ pieces.

### MIC determinations

The MIC was assayed according to the procedures outlined by the Clinical and Laboratory Standards Institute (CLSI) [27]. MIC was taken as the lowest drug concentration at which observable growth was inhibited. Experiments were performed in triplicate.

### Hemolytic Activity

Murine erythrocytes were diluted to 6.5–7 × 10^7^ cells per ml, and 25 μl aliquots were added to 2 ml of sodium phosphate-buffered saline (PBS) (8.1 mM phosphate, pH 7) solution, pH 7.3, with or without FS10. As control 1 ml of melittin was added to 1 ml of blood suspension and incubated at 37°C for 1 h. The samples were then centrifuged at 1500xg for 10 min, and the supernatant absorbance was measured at 414 nm. Total hemoglobin was determined by suspending the same aliquot of cells in 2 ml of distilled water and measuring the absorbance at 414 nm. All the experiments were performed in triplicate.

### Preparation of inoculum

Bacteria were grown in brain-heart infusion broth (Sigma Aldrich Milan, Italy). When bacteria were in the log phase of growth, the suspension was centrifuged at 1000 x g for 15 min, the supernatant discarded, and the bacteria were resuspended and diluted into sterile saline to achieve a concentration of approximately 5x10^7^ CFU/ml.

### Mouse wound infection model

The study included a total of 144 animals divided into 12 groups (each composed of 12 mice). For each strain, the study included a control group without infection (C_0_), a control infected group that did not receive any treatment (C_1_ and C_2_), a control infected group with drug-free Allevyn (containing 20 μg FS10) (C_3_ and C_4_) and three infected groups treated, respectively, with: i) FS10-soaked Allevyn (T_1_ and T_2_), ii) daily intraperitoneal tigecycline (7 mg/Kg) and drug-free Allevyn (T_3_ and T_4_), iii) FS10-soaked Allevyn (containing 20 μg FS10) and daily intraperitoneal injections of tigecycline (7 mg/Kg) (T_5_ and T_6_).

The main outcome measures were quantitative culture and histological examination of tissue repair. Mice were anesthetized by an intramuscular injection of ketamine (50 mg/kg of body weight) and xylazine (8 mg/kg of body weight) hair on the back was shaved and the skin cleansed with 10% povidone-iodine solution. Using a 1x2 cm template, one full thickness wound was established, by clamping and cutting with scissors, through the panniculus carnosus on the back subcutaneous tissue of each animal. A small gauze was placed over each wound and then inoculated with 5x10^7^ CFU of *S*. *aureus* ATCC 43300 or (MS) *S*. *aureus* ATCC 29213. The pocket was closed by means of skin clips [28]. This procedure resulted in a local abscess at 24 h. One wound was created per animal. The animals were returned to individual cages and thoroughly examined daily. After 24 h, the wound was opened in control animals; the gauze was removed for quantitative bacterial culture and also plated onto sheep blood agar in order to determine if other bacterial species were present. Treatment was initiated in infected animals and intra-peritoneal Tigecycline (7 mg/Kg) was administered daily for 7 days, while topical treatment (FS10 and Allevyn with or without tygecicline) was applied every 2 days. Peptide absorption was generated immediately before implantation by soaking Allevyn for 20 minutes in a sterile FS10 solution (10 mg/l), resulting in 20 μg FS10 on the 1x2 cm^2^ Allevyn.

Animals, at day 8 post-wounding, were euthanized and a 1x2 cm area of skin, including the wound, was excised aseptically. Skin samples were divided into two. One piece was used for histological examination (see below) and the other was weighted and then homogenized in 1 ml PBS using a stomacher. Quantitation of viable bacteria was performed by culturing serial dilutions (0.1 ml) of the bacterial suspension on blood agar plates. All plates were incubated at 37°C for 48 hours and evaluated for the presence of bacteria. Counting the number of CFUs per plate quantitated the organisms. The limit of detection for this method was approximately 10 CFU/g. Toxicity was evaluated on the basis of the presence of any drug-related adverse effects, behavioral alterations and local signs of inflammation.

### Histological examination

In order to assess at histological level the effects of different treatment modalities on the healing process of infected wounds, samples were surgically removed from wounds at the time of euthanasia and were frozen in liquid nitrogen and stored at –70°C. Five μm tissue sections, including the epidermis, the dermis, and the subcutaneous panniculus carnosus muscle, were cut by a microtome, air-dried overnight and then fixed in acetone for 10 min. The sections were then stained with haematoxylyn and eosin. An observer blinded to treatment performed all subsequent analyses. The specimens were observed under light microscopy to evaluate visible morphological differences in the character of wound healing among the categories. A five-tiered grading system based on epithelial presence, degree of stratification, degree of differentiation as well as maturational features of the granulation tissue was devised to capture and report these differences among the control and treatment groups (score system in table 1). Scores were given in accordance with a system reported previously to evaluate maturity of wound repair [12].

**Table 1:** A five-tiered grading system to evaluate maturity of wound repair.

Score	Re-epithelialization	Granulation tissue	Collagen deposition
0	Trace and focal migrating	Trace	None
1	Migrating	Hypocellular and none vessel	Trace
2	Partial stratum corneum	Many cells and few vessels	Slight
3	Hypertrophic and partial stratum corneum	Many fibroblasts, some fibers and some vessels	Moderate
4	Complete and normal	More fibers, few cells	Marked

### Statistical analysis

Quantitative evaluations of the bacteria in excised tissue were presented as means ± standard deviations (SDs); statistical comparisons between groups were made by analysis of variance (ANOVA). Statistical analysis of the score of morphological features related to wound repair between groups was performed by Bonferroni’s test. Significance was accepted when the P value was <0.05.

## Results

### Susceptibility testing

The staphylococcal isolates showed susceptibility to tigecycline, that exhibited MICs of 0.12 and 0.25 mg/l for MSSA and MRSA strains. Finally, FS10 did not demonstrate any in vitro activities against the two strains (MICs >256 mg/l).

### Hemolytic activity

To evaluate a possible toxic effect of FS10 on animals related to its hemolytic activity, peptide ability to disrupt mice erythrocyte membranes was assessed in comparison with melittin. FS10 was significantly less effective than melittin in permeabilizing erythrocytes. Even at a higher concentration tested, a cellular disruption inferior to 8% was observed.

### Quantitative culture of excised tissues

**Fig 2:**
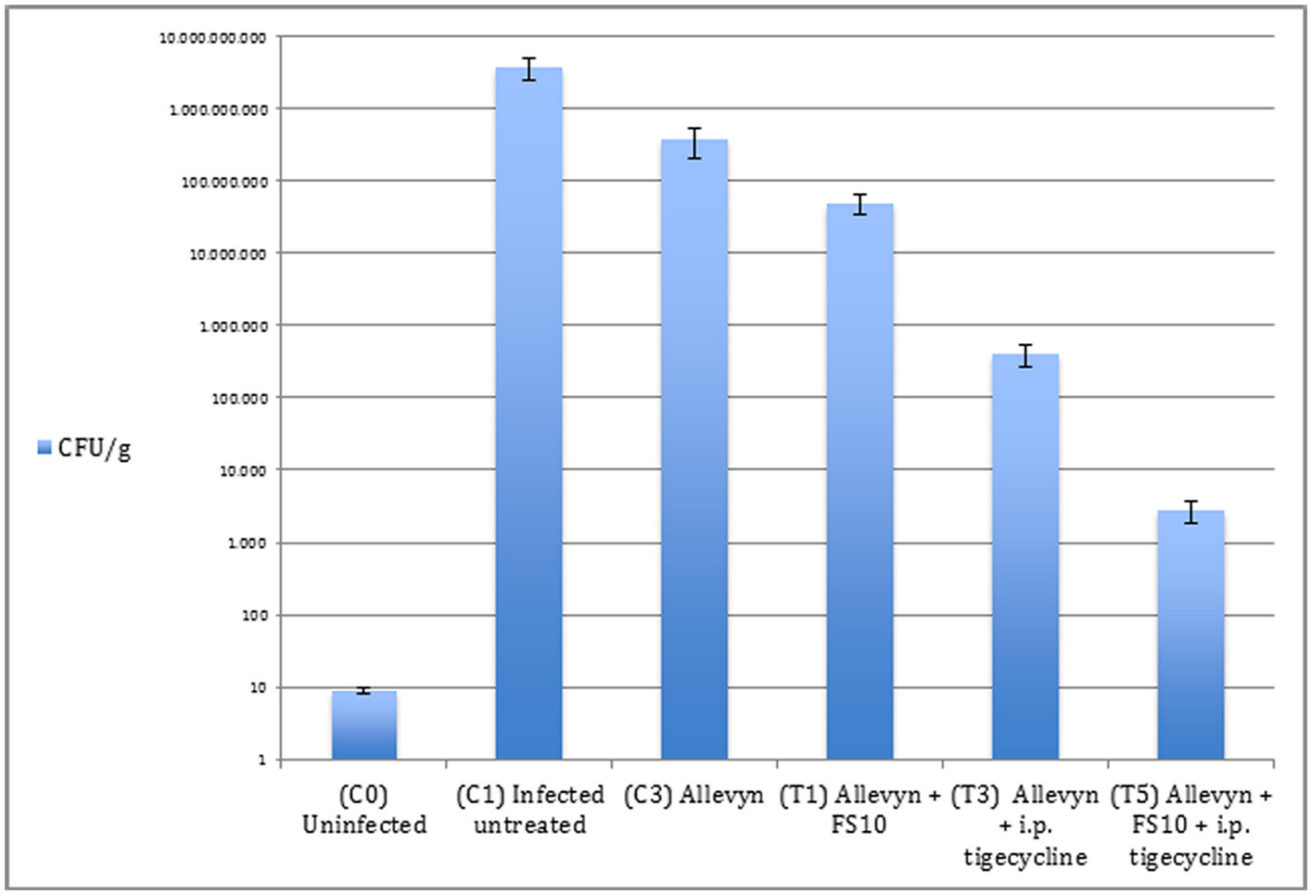
Quantitative cultures observed in MSSA infection. The limit of detection for this method was approximately 10 CFU/g. ANOVA test p<0.05: C_3_ vs C_1_; p<0.05 T_1_, T_3_, T_5_ vs C_1_; p<0.005 T_5_ vs T_3_ and T_1_

Overall, data analysis showed that inhibition of bacterial growth was achieved in all treated groups. In details for the MSSA strain, mean bacterial numbers in untreated controls (3.8 x 10^9^ ± 1.3 x 10^9^ CFU/g) were significantly higher than those recovered from all treatment groups (Fig 2). Treatment with FS10 alone had a slight effect, reducing the mean bacterial numbers to 4.9 x 10^7^ ± 1.5 x 10^7^ CFU/g). Tigecycline had a strong effect compared with control infected untreated animals and FS10 treated group (4.0 x 10^5^ ± 1.3 x 10^5^ CFU/g). The highest inhibition of infection was achieved in the group that received FS10-soaked Allevyn and parenteral tigecycline reducing bacterial number to 2.8 x 10^3^ ± 1.0 x 10^3^ CFU/g (p<0.005)(Fig 2).

**Fig 3:**
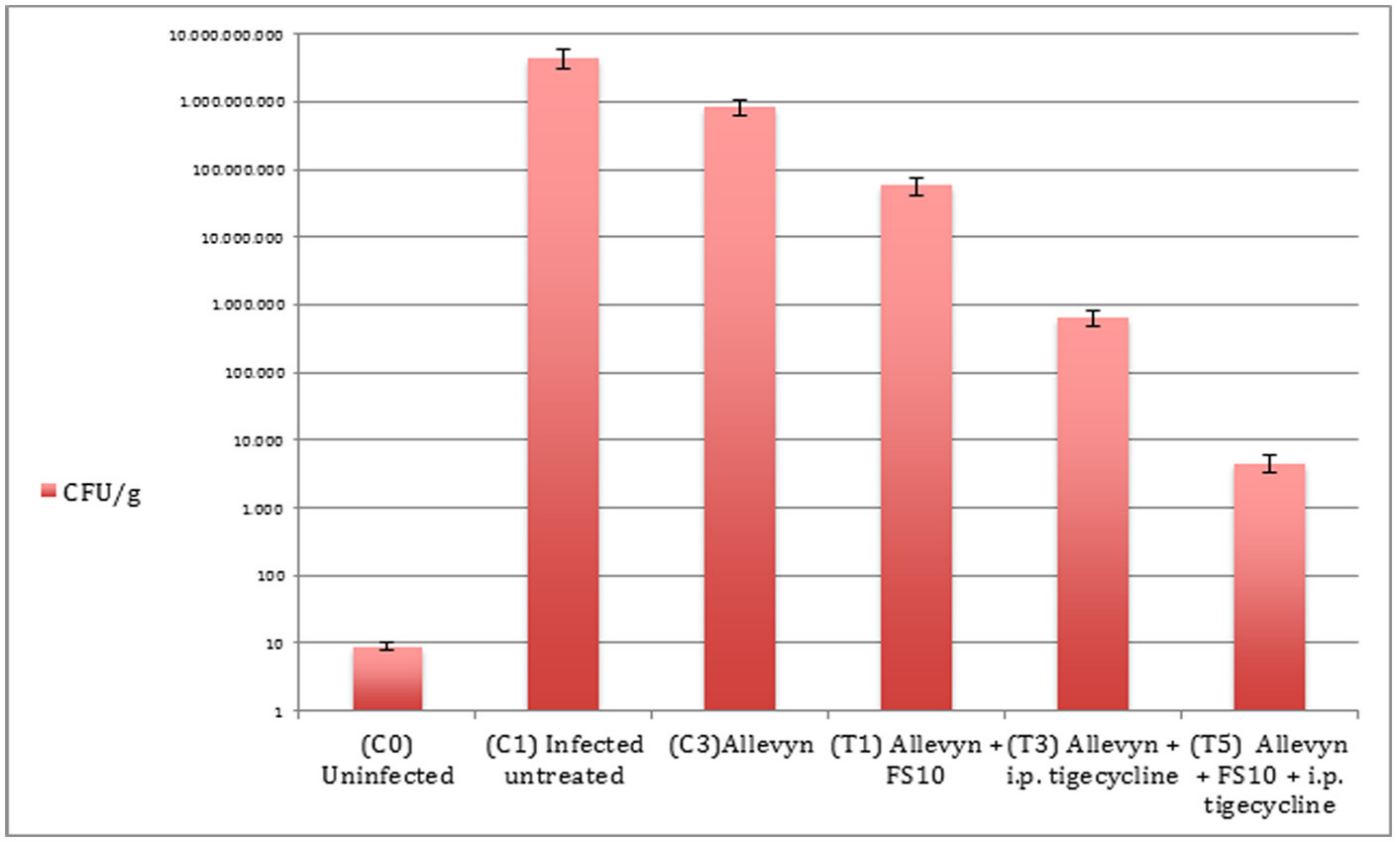
Quantitative cultures observed in MRSA infection. The limit of detection for this method was approximately 10 CFU/g. ANOVA test p<0.05 C_4_ vs C_2_; p<0.005 T_2_, T_4_, T_6_ vs C_2_; p<0.005 T_6_ vs T_4_ and T_2_

Comparable results were observed for the MRSA strain. In particular, mean bacterial numbers in untreated controls (4.5 x 10^9^ ± 1.4 x 10^9^ CFU/g) were significantly higher compared to the all treatment groups (Fig 3). FS10-soaked Allevyn showed 3.7 x 10^8^ ± 1.6 x 10^8^ CFU/g. Tigecycline had a strong effect when compared with control untreated animals or group treated with FS10 (6.4 x 10^5^ ± 1.7 x 10^5^ CFU/g). The highest inhibition of contamination was achieved in the group that received FS10-soaked Allevyn and systemic tigecycline, reducing bacterial number to 4.6 x 10^3^ ± 1.3 x 10^3^ CFU/g (p<0.005)(Fig 3).

None of the animals included in any group died or had clinical evidence of drug related adverse effects, behavioral alterations and local signs of inflammation.

### Histological evaluation of excised tissues

Since the microbiological results were similar in the both strain, we decided to perform histological evaluation only in the group infected with MRSA that is representative of a clinical hospitalized infection. The impact of different treatment regimens on wound healing was scored and summarized in Tables 1 and 2.

**Table 2:** Summary of the biological impact of different treatment on wound healing parameters at day 8 post-wounding in MRSA mice models.

MICE MODEL	EPITHELIALIZATION Score (mean±S.D.)	GRANULATION TISSUE (mean±S.D.)	COLLAGEN ORGANIZATION (mean±S.D.)
C0	3.30±035	3.28±0.65	3.10±0.58
C2	0.4±0.15	0.35±0.15	0.30±0.20
C4	0.65±0.30	0.75±0.28	0.50±0.28
C5	3.40±0.45	3.10±0.78	3.20±0.50
T_2_	2.80±0.30	2.80±0.50	3.0±0.20
T_4_	3.00±0.45	2.90±0.20	3.0±0.10
T_6_	3.50±0.20	3.25±0.40	3.20±0.15

(C0) Control group (non-infected mice); (C2) MRSA-infected mice without treatment; (C4) MRSA-infected mice with drug-free-Allevyn; (C5) Non infected mice with drug-free Allevyn; (T2) MRSA-infected mice treated with Allevyn and FS10; (T4) MRSA-infected mice treated with Allevyn and tigecicline; (T6) MRSA-infected mice treated with Allevyn tigecicline and FS10.

Bonferroni test p<0.05

C_0_, C_5_, T_2_,T_4_ *vs* C_2_,C_4_

C_0_,C_5_,T_2_,T_4_,T_6_ *vs* C_2_,C_4_

T6 *vs* C0,C2,C4,T2

In the present study, we investigated the efficacy of FS10, alone or together with Allevyn and tigecycline, in a mouse model of surgical wound infection.

**Fig 4:**
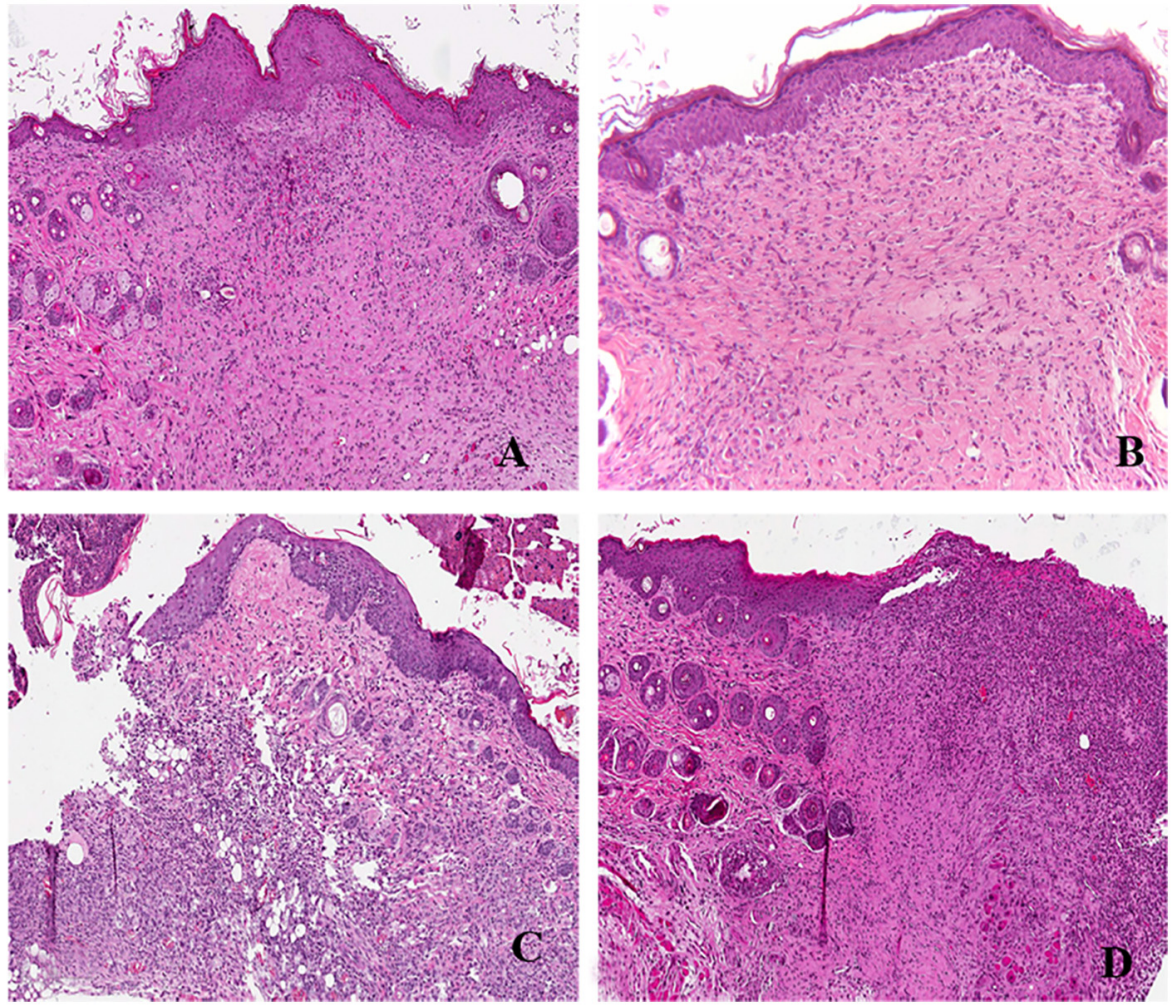
Histology of mouse wound healing at day 7 after injury. A) Non infected mice; B) MRSA-infected mice without treatment; C)MRSA-infected mice with Allevyn; D)Non infected mice with Allevyn. (Hematoxylin and Eosin; original magnification x200).

We considered as a control a biopsy of healthy skin (not infected mice) (C_0_)(average epithelialization score 3.30, granulation score 3.28 and collagen organization score 3.10) (Fig 4A).

Similar results were observed in not-infected mouse skin wounds with drug-free Allevyn (C5) with an average epithelialization score of 3.40, granulation tissue score of 3.10 and collagen organization score of 3.20 (Fig 4B). On the contrary untreated MRSA-infected mice (C2) and MRSA-infected wounds with Allevyn (C4) displayed the worst healing process among all groups considered (p<0.05) with average epithelial/granulation/collagen score of 0.4/0.35/0.30 and 0.65/ 0.75/0.50 respectively (Fig 4C and 4D).

**Fig 5:**
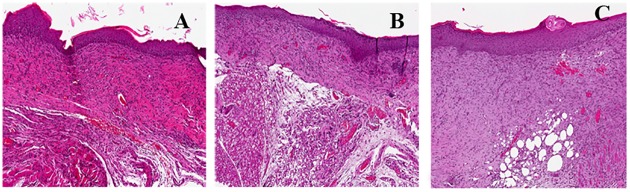
Histology of mouse wound healing at day 7 after injury. A) MRSA-infected mice treated with Allevyn and FS10; B) MRSA-infected mice treated with i.p.tigecycline; C)MRSA-infected mice treated with tigecycline and FS10. (Hematoxylin and Eosin; original magnification x200).

MRSA-infected wounds treated with Allevyn + FS10 (T2) and MRSA-infected wounds treated with drug free Allevyn + tigecycline (T4) displayed similar healing scores. In fact in T2 we observed an average epithelialial/granulation score of 2.80 and collagen organization score of 3.0; in T4 epithelial/ collagen score was 3.0 and granulation tissue score was 2.90. (Fig 5A)

In MRSA-infected wounds treated with Allevyn + tigecycline + FS10 (T6) we observed a better overall healing (average epithelialization score 3.50, granulation score 3.25 and collagen score 3.20 (Fig 5B and 5C). In particular epithelialization and collagen scores were significantly higher than those recovered from all treatments groups (p<0.05).

## Discussion

Increased incidence of MRSA-associated infections in both acute and chronic wounds is well-documented and it could be explained by an increase in general life expectancy, overall morbidity, and the use of advanced medical interventions that require invasive devices, like indwelling catheters and prosthetic devices [29].

As biofilm formation has been shown to be a mechanism for evading host-defenses and resisting the effect of antimicrobials, it has been suggested that strains capable of forming biofilms may persist within the host contributing to relapsing/chronic infections [30–31]. It has been observed that treatments, which specifically target biofilms, transformed non-healable wounds into healable ones, suggesting that the use of suitable topical agents that inhibit biofilm formation and/or disrupt preformed biofilm should be an integral part of the management of chronic wound infections. Chronic wounds represent a silent epidemic that affects a large fraction of the world population and poses a major and gathering threat to the public health and economy [2].

In order to investigate functional consequences of staphylococcal infections on wound healing we utilized a well-established mouse skin wound model [8, 11, 15, 17]. We decided to study two staphylococcal strains, with different susceptibility, to demonstrate that efficacy of FS10 alone and in combination with tigecycline works independently of the studied strains. Moreover, the utility and translational validity of animal models in research has recently been discussed [32, 33]. When evaluating the potential clinical utility of a new topical treatment, animal models provide an important link between in vitro susceptibility findings and anticipated clinical results, and help to identify targets for the development of novel treatment and prevention strategies [34].

To date, a great deal of effort has been dedicated to the discovery of small molecules able to interfere with the quorum sensing system, which controls the expression of several virulence factors and influences bacterial biofilm growth and development. Quorum sensing signaling can be modulated by autoinducing molecules such as N-acyl homoserine lactones, oligopeptides and autoinducer-2 [35]. Gram positive bacteria, like *S*. *aureus*, use linear or cyclic peptides as their autoinducer signals. RIP, a peptidic inhibitor of QS produced by *S*. *aureus*, represents a valuable candidate for drug discovery since it is able to inhibit *S*. *aureus* virulence and biofilm formation by a mechanism which is still unclear. FS10, which corresponds to a truncated sequence of RIP, could compete with RNAIII activating protein (RAP), just like RIP, for the activation of TRAP (targeting of RNAIII-activating peptide) and consequently inhibiting *agr* quorum sensing. Like many short peptides, FS10 does not possess a fixed conformation in solution, but the presence of proline in P2 as a conformational constraint could predefine a less flexible sequence that well fits the binding site of RNAIII-inhibiting enzyme resulting in an inhibitor.

In our study, tigecycline, in both strains, had a stronger effect on bacterial numbers in infected treated animals compared to controls or FS10 treated group, although the highest inhibition of infection was achieved in the groups that received FS10-soaked Allevyn and parenteral tigecycline. In previous investigations quorum sensing inhibitors have already shown their capacity for a positive interaction with commonly used antibiotics [20–21]. It can be hypothesized that the reduction of high titers bacteria, achieved with antibiotics coupled to a less pathogenic property by the remaining circulating bacteria obtained with the inhibition of quorum sensing, reduced the onset of infection and improved microbiological data.

Wound healing is a complex well-orchestrated process with overlapping phases of hemostasis, inflammation, proliferation, and remodeling [30]. Hemostasis occurs within minutes to hours of injury with the formation of a provisional matrix and initiation of the inflammatory phase, that in adult wound healing is exuberant and is constituted particularly by neutrophilic granulocytes and macrophages within the wound bed after injury. The proliferative phase overlaps with inflammation, with the formation of granulation tissue followed by immigration of fibroblasts into the wound bed and re-epithelialization by keratinocytes [25, 36].

Our data showed an evident wound healing delay in infected untreated mice, with poor re-epithelialization, complete absence of epithelium, persistence of abundant inflammatory infiltrate, reduced accumulation of granulation tissue with many cells and some fibers, and a slight deposition of collagen. Conversely, in all the treated groups, the process of healing orderly progressed after injury, with variable degrees of re-epithelialization, enlargement of granulation tissue, and deposition of collagen. In particular, infected mice, receiving FS10 showed a slight and focal keratinization, while the granulation tissue showed many cells and some vessels. Moreover an evident inflammatory infiltrate was still present, while the collagen fibers were organized but still not regular. The group treated with tigecycline and FS10-soaked Allevyn showed robust epidermal coverage, with a reconstitution of the regular epidermal lining and an evident keratinization, although the dermal papillae were still few. Thick granulation tissue with many vessels and fibers was present, with a sensible reduction of fibrinous exudation and a higher score for collagen, in terms of more organized and regular collagen fibers compared to those seen in the other treatments. Also we did not observe an evident inflammatory response.

## Conclusions

The present study shows that combinations of topical FS10 with i.p. tigecycline induced positive interaction in vivo, resulting in enhanced therapeutic benefit versus systemic MRSA infections in murine models. This represents, indeed, a novel route to target MRSA infections which is worthy of further investigation. Moreover, we observed that the effectiveness of FS10 combined to tigecycline treatment may not only be due to a direct action on quorum sensing but also to the stimulation of wound healing process, as observed in our previous study [25]. Finally, this study demonstrated the utility of the mouse model as an inexpensive, rapid in vivo model to pre-screen potential novel antimicrobial therapies prior to more extensive testing.
